# Hyperammonemia due to urea cycle disorders: a potentially fatal condition in the intensive care setting

**DOI:** 10.1186/2052-0492-2-22

**Published:** 2014-03-13

**Authors:** Marcel Cerqueira Cesar Machado, Fabiano Pinheiro da Silva

**Affiliations:** Emergency Medicine Department, University of Sao Paulo, Sao Paulo, 05508-070 Brazil; Faculdade de Medicina, USP, Av Dr Arnaldo 455 Room 3189 LIM 51, Sao Paulo, 05508-070 Brazil

**Keywords:** Hyperammonemia, Neurological disorders, Intensive care unit

## Abstract

Disorders of the urea cycle are secondary to a defect in the system that converts ammonia into urea, resulting in accumulation of ammonia and other products. This results in encephalopathy, coma, and death if not recognized and treated rapidly. Late-onset urea cycle disorders may be precipitated by acute disease and can be difficult to recognize because patients are already ill. Diagnosis of urea cycle disorders is based on clinical suspicion and determination of blood ammonia in suspected patients with neurological symptoms in the intensive care setting. Treatment is based on the removal of ammonia by dialysis or hemofiltration, reduction of the catabolic state, abolishment of nitrogen administration, and use of pharmacological nitrogen scavenging agents.

## Introduction

Recently, we reported a case of a 49-year-old man with biliary acute pancreatitis who developed high fever and a large pancreatic abscess. Upon admission, this patient underwent necrosectomy, cholecystectomy, and abscess drainage in a single surgical setting. Parenteral nutrition was initiated, but on the 8th day, the patient developed progressive lethargy, confusion, and coma, requiring intubation. Computed tomography and magnetic resonance imaging showed no brain lesions. Laboratory tests indicated normal liver function, with enzyme levels and serum bilirubin within the normal range. The etiology of the encephalopathy was not suspected until hyperammonemia (137 μ/L) was observed. Lactulose treatment produced no improvement in clinical signs, and the patient remained in a deep coma, with ammonia levels elevated up to 254 μ/L. Because there was no evidence of liver disease, hyperammonemia must have been caused by a metabolism disorder. To deal with these types of cases, physicians need to understand the principles of the pathophysiology of ammonia metabolism and how to treat these patients in the intensive care setting.

## Review

### Ammonia metabolism

**Figure 1:**
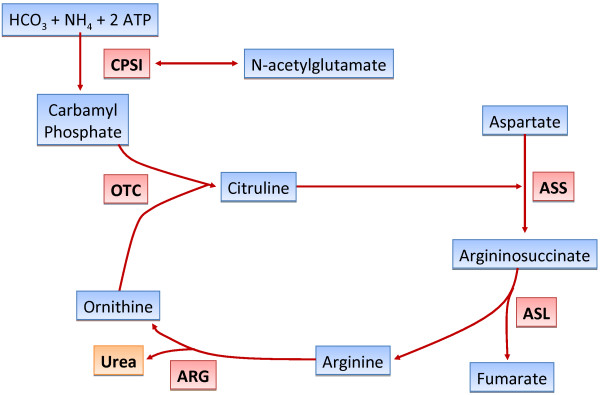
**The urea cycle.** *CPS-1* cabamoyl phosphate synthetase 1, *OTC* ornithine transcarbamylase, *ASS* argininosuccinic acid synthetase, *ASL* argininosuccinic acid lyase, *ARG* arginase.

The urea cycle is the only effective system that converts waste nitrogen from protein intake and the breakdown of endogenous protein (catabolism) into urea, which is excreted from the body. This system consists of five consecutive enzymatic reactions that convert one molecule of nitrogen from ammonia, two molecules of nitrogen from ornithine, and one molecule from aspartate to urea in each cycle (Figure 1). There is also a cofactor-producing enzyme (*N*-acetyl glutamate synthetase) and two transporters (ornithine transporter-1 and citrin) that are involved in the urea cycle.

Disorders of the urea cycle are the result of total or partial deficiency in any of the factors mentioned (Figure 1). This leads to defects in the metabolism of waste products from breakdown of protein and other nitrogen-containing substances, with accumulation of ammonia and other products [1, 2]. The most common presentation of these defects is in newborns who typically present with somnolence, poor feeding, hyperventilation, and seizures, followed by lethargy and coma [3]. However, there are several reports concerning urea cycle disorders (UCDs) that manifest in adulthood, mainly in the presence of an acute injury [2]. In most cases in the intensive care setting, underlying UCDs are difficult to recognize because patients are in a critical condition for other reasons. There is also an insufficient awareness of medical staff because of the rarity of this disease and because the plasma ammonia levels are not routinely determined. Prompt diagnosis and treatment of this disorder are required to avoid death or severe neurological damage.

In the last few decades, there has been a dramatic improvement in the recognition and treatment of UCDs [3–7]. In partial deficiency of urea cycle enzymes, symptoms may develop late in life. These symptoms are frequently triggered by acute stress or illness, such as sepsis, surgery, trauma, or drugs (e.g., valproic acid), because of their effect on the function of the enzyme carbamoyl phosphate synthetase-1 (CPS-1) [2] especially in patients on parenteral nutrition [8]. In these situations, elevation of blood ammonia and symptoms may be subtle or occur acutely only during metabolic decompensation related to catabolic stress, such as in surgery, sepsis, or hemodynamic alterations. Recognition and treatment are crucial to improve the outcome of UCDs. Ammonemia may be normal when an individual is healthy. We present here a review of the clinical features, diagnosis, and treatment of these disorders in late-onset diseases related to partial enzyme deficiency decompensated by acute stress.

### Partial UCDs or late-onset UCDs

Patients with partial deficiency of any enzyme in the urea cycle may develop symptoms at any time of life, usually as a result of increased catabolic stress [9, 10]. Partial enzyme deficiency allows a patient to have a normal life for decades before there is increased production of ammonia or interference with any enzyme of the urea cycle. In patients with partial urea cycle enzyme deficiency, symptoms may be delayed for months or years and vary with the specific enzyme deficiency. In most hyperammonemic episodes, patients may present with a loss of appetite, vomiting, lethargy, and behavior abnormalities associated with hallucinations, sleep disorders, ataxia, and even seizures. These episodes are usually related to periods of high protein intake, systemic infection, or catabolic stress. In the intensive care unit, patients with acute neurological and psychiatric symptoms or coma should have plasma ammonia levels measured to disclose a possible late-onset UCDs if there is any suspicion. Prompt diagnosis of UCDs is essential for treatment to be effective; otherwise, the prognosis of these patients is poor. UCDs are usually difficult to recognize because many of these patients are frequently ill for other reasons, such as sepsis, acute pancreatitis, trauma, or other acute diseases. Plasma ammonia determination is carried out in venous or arterial blood collected in chilled tubes with ammonia-free sodium heparin or EDTA tubes placed on ice. These are immediately sent to the laboratory because hemolysis, an increase in temperature, and delays in processing may falsely increase plasma ammonia levels (normal levels less than 30 μ/L). An elevated plasma ammonia level is associated with normal blood glucose levels. A normal anion gap suggests UCDs because hyperammonemia associated with hyperglycemia and an abnormal anion gap are found in organic acidemias (a different inborn error of metabolism). Plasma and urine must be frozen for future diagnostic tests to identify the specific enzyme deficiency. The following are the conditions that may induce hyperammonemia in partial UCDs:

1. Postpartum stress
2. Parenteral nutrition with high protein administration
3. Gastrointestinal bleeding
4. Administration of valproic acid
5. Infection and postoperative stress

### Neurological disorders in UCDs

Hyperammonemia may damage the brain by a variety of mechanisms. The most important mechanism is brain edema, probably induced by disruption of the aquaporin system (membrane water channels) and brain electrolyte homeostasis. The central feature of hyperammonemia-induced encephalopathy is an increase in astrocytes glutamine synthesis, and swelling of astrocytes in response to the osmotic effect of glutamine accumulation, resulting in increased intracranial pressure [11]. A recent study using *N*-ammonia positron emission tomography suggested that the magnitude of the flux of ammonia from the blood into intracellular glutamine in the brain is primarily correlated with blood ammonia concentrations [12]. In infancy, brain damage in acute hyperammonemia is similar to that seen in hypoxic-ischemic situations, with white matter disruption and lacunar infarcts. Brain damage due to hyperammonemia may result in seizures and coma [13]. Subclinical seizures are common in acute hyperammonemia and may be considered in the treatment of these conditions. In many cases, modern imaging techniques may provide information related to the extent and intensity of brain injury. However, routine neurological images may not detect brain damage [14]. Newer technologies, such as magnetic resonance spectroscopy, diffusion tensor imaging, and functional magnetic resonance may provide information about the many types of neurological damage seen in UCDs.

### Therapy of a rapid decrease in plasma ammonia concentrations to normal levels

In patients, including the one we recently reported, with high ammonia plasma levels higher than 100 μ/L, the first option is to decrease plasma ammonia levels by using an extracorporeal therapy to achieve a rapid reduction in plasma ammonia levels. Renal replacement therapy (RRT) allows efficient removal of toxic metabolites, minimizing the duration of the metabolic disturbance. The use of RRT has changed since the studies of Donn et al. [15], concluding that hemodialysis is the preferred modality for the treatment of hyperammonemia secondary to UCDs. Currently, the choice of treatment for hyperammonemia is hemodialysis or continuous RRT, or even both modalities as early as possible [16–18]. The clearance of ammonia and other low-molecular-weight toxins is much greater with hemodialysis than with other RRTs [16, 17, 19]. Hemodialysis is the first-line therapy for the initial treatment of hyperammonemia in UCDs. Because ammonia is a gas, its rapid removal by hemodiafiltration is not associated with osmotic problems, and no special care must be taken to avoid dialysis disequilibrium syndrome [17, 18]. Peritoneal dialysis is not effective for this purpose and should not be used. Hemodialysis may be discontinued when plasma levels fall below 80 μ/L. However, clinical evaluation is the best parameter to be used for discontinuation of dialysis. Patients with severe encephalopathy from acute hyperammonemia may completely recover unless prolonged cerebral edema results in cerebral damage [11]. In fact, in our patient, 36 h after continuous hemofiltration, ammonia levels had decreased to 82 μ/L, with improvement in the patient's mental status. The patient was weaned from the respirator and became completely awake. Rebound hyperammonemia with clinical worsening may also occur, requiring further dialysis. In our patient, discontinuation of hemofiltration was accompanied by deterioration in the patient's mental status. Any oral or parenteral protein administration must be immediately discontinued when hyperammonemia is detected.

### Pharmacological treatment

#### Nitrogen scavenger therapy

Sodium phenylacetate and sodium benzoate, or sodium phenylbutyrate, are available for intravenous or oral administration. The basis for use of these drugs was established by Brusilow et al. [20] in 1979, who proposed the use of alternative pathway treatment to reduce ammonia plasma levels in UCDs. Alternative pathway treatment diverts nitrogen from the urea cycle to alternative routes of excretion. Sodium phenylacetate combines with glutamine, producing phenylacetylglutamine. Phenylacetylglutamine is excreted by the kidneys and sodium benzoate conjugates with glycine, producing sodium hippurate, which is also excreted by the kidneys [21].

#### Replacement of deficient urea cycle intermediates

Arginine administration in patients without a definitive diagnosis of the specific type of UCD is also important because ornithine transcarbamylase (OTC) is the most common type of late-onset UCDs [2, 20, 22]. Low plasma arginine levels observed in patients with OTC deficiency may also be related to increased vascular thrombosis [23].

To prevent future episodes of hyperammonemia, plasma ammonia should be maintained below 80 μ/L, glutamine below 1,000 μ/L, and arginine between 80 and 150 μ/L [14]. In our patient, we administered sodium benzoate (3 g) and arginine (3 g) every 4 h via a nasogastric tube, maintaining plasma ammonia levels within the normal range. Recently, it was found that glycerol phenylbutyrate, which has excellent pharmacokinetics, controls plasma ammonia, improving executive function in adult and pediatric patients [24].

### Reduction of protein catabolism

Restriction of protein intake for a period of 24 to 48 h and administration of calories from glucose and fat are important in patients on hemodialysis or hemofiltration for the prevention of an excessive catabolic state. Maintenance of adequate plasma levels of essential amino acids is also necessary to avoid the catabolic state. Low-dose continuous infusion of insulin with glucose may be used to potentially alleviate the catabolic state.

### Liver transplantation

Liver transplantation is considered only in patients with recurrent hyperammonemia or in those resistant to conventional medical therapy. The decision for liver transplantation is also based on the extent of brain and liver damage.

### Diagnosis, genetic tests, and differential diagnosis of UCDs

The elevation in plasma ammonia concentrations is the main alteration in UCDs. Normal ammonia levels in adults are less than 30 μ/L. Quantitative plasma amino acid concentrations are used to determine specific enzyme deficiency in UCDs (Table 1). Plasma levels of citrulline can be used to evaluate the type of UCD and to separate proximal from distal urea cycle defects. Plasma citrulline levels are absent or present in trace amounts in CPS-1 deficiency and in low, or even normal, concentrations in late onset of OTC deficiency. Plasma citrulline levels are present in high concentrations (tenfold) in argininosuccinic acid synthetase deficiency. Moderate elevation in plasma citrulline levels is seen with argininosuccinic acid lyase deficiency, accompanied by an elevation in argininosuccinic acid in plasma and urine (Table 1). Plasma concentrations of arginine are reduced in every type of UCD, except in arginase deficiency (elevation five to sevenfold). In late onset or in partial enzyme defects, arginine concentrations may be normal. Plasma concentrations of glutamine, asparagine, and alanine are elevated in UCDs.

**Table 1:** **Analysis of plasma amino acids and urinary orotic acid**

	CPS-1 deficiency	OTC deficiency	ASS-1 deficiency	ASL deficiency
Citrulline	Absent	Low	Elevated (tenfold)	Elevated (twofold)
Arginine	Reduced	Reduced	Reduced	Reduced
Orotic acid	Low	Elevated	Elevated	Elevated

Urine concentrations of orotic acid help to distinguish CPS-1 deficiency from OTC deficiency because they are normal or low in CPS-1 deficiency and elevated in OTC deficiency. Urinary orotic acid concentrations are also elevated in arginase deficiency and in citrullinemia type 1 deficiency (Table 1). Diagnosis may be further refined by enzyme analysis in appropriate tissues as follows: liver biopsy (CPS-1, *N*-acetyl glutamate synthetase, and OTC deficiencies); red blood cells (arginase deficiency); and fibroblasts (skin biopsy, argininosuccinic acid synthetase and argininosuccinic acid lyase deficiencies).

### Genetic tests

There are several genetic tests available for the diagnosis of UCDs. In late-onset hyperammonemia, DNA testing for OTC deficiency should be the first test for evaluation because this is the most common type of late-onset UCD, and many mutations have been described in this condition. Recently, a more sophisticated DNA mutation analysis has been introduced, which allows identification of variants in most coding genes [25].

### Differential diagnosis of UCDs

Several disorders disturbing liver function may mimic UCDs. These include hepatic encephalopathy in patients with advanced liver disease, vascular bypass of the liver, valproic acid or cyclophosphamide poisoning, herpes simplex infection, and gastrointestinal bacterial overgrowth. In the last two conditions, the plasma ammonia levels are usually moderately elevated. A number of inborn errors of metabolism may also cause hyperammonemia:

- ● Tyrosinemia type 1
- ● Galactosemia
- ● Mitochondrial disorders
- ● Fatty acid oxidation disorders
- ● Citrin deficiency leading to citrullinemia type II (CTLN2) and neonatal intrahepatic cholestasis caused by citrin deficiency (NICCD)

Citrin deficiency (CTLN2) is a late-onset disorder characterized by recurrent periods of hyperammonemia, delirium, irritability, and liver fatty infiltration, but there is no hepatic dysfunction. Citrin is an aspartate glutamate transporter across the mitochondrial membrane [2]. Citrin deficiency leads to a decrease in cytoplasmic aspartate, limiting the activity of the enzyme argininosuccinic acid synthase [2].

Diagnosis of this alteration is based on findings of hyperammonemia and increased plasma concentrations of citrulline and arginine. SLC25A13 is a gene mutated in patients with citrin deficiency. Ornithine translocase deficiency is a rare disease that results in hyperornithinemia, homocitrullinuria, and hyperammonemia, similar to those in UCDs. A reduction in ornithine transport into the mitochondria results in orotic aciduria and deficiency of urea synthesis. In this situation, plasma levels of ornithine are high and can be reduced by a low-protein diet. Citrin deficiency and hyperammonemia may be included in UCDs as transporter defects. Therefore, UCDs is related to eight defects, with deficiencies in six enzymes and two transporters.

## Conclusions

Hyperammonemia in an acutely ill patient can cause irreversible neurological damage, or even death, if not recognized. Determination of plasma ammonia levels should be included in a laboratory work-up in case of acute psychiatric and neurological symptoms or unexplained coma in the intensive care unit.
